# Dataset Reuse: Toward Translating Principles to Practice

**DOI:** 10.1016/j.patter.2020.100136

**Published:** 2020-11-04

**Authors:** Laura Koesten, Pavlos Vougiouklis, Elena Simperl, Paul Groth

**Affiliations:** 1 King's College London, London WC2B 4BG, UK; 2 Huawei Technologies, Edinburgh EH9 3BF, UK; 3 University of Amsterdam, Amsterdam 1090 GH, the Netherlands

**Keywords:** dataset reuse, human-data interaction, reuse prediction, neural networks, data portals

## Abstract

The web provides access to millions of datasets that can have additional impact when used beyond their original context. We have little empirical insight into what makes a dataset more reusable than others and which of the existing guidelines and frameworks, if any, make a difference. In this paper, we explore potential reuse features through a literature review and present a case study on datasets on GitHub, a popular open platform for sharing code and data. We describe a corpus of more than 1.4 million data files, from over 65,000 repositories. Using GitHub's engagement metrics as proxies for dataset reuse, we relate them to reuse features from the literature and devise an initial model, using deep neural networks, to predict a dataset's reusability. This demonstrates the practical gap between principles and actionable insights that allow data publishers and tools designers to implement functionalities that provably facilitate reuse.

## Highlights

• A compilation of reusability features of datasets from literature
• A corpus of 1.47 million datasets from 65,537 repositories source from GitHub
• A case study on GitHub using a five-step approach to understand projected data reuse
• A machine learning model that helps to predict dataset reuse in the case of GitHub

## The Bigger Picture

The web provides access to millions of datasets. These data can have additional impact when it is used beyond the context for which it was originally created. We have little empirical insight into what makes a dataset more reusable than others, and which of the existing guidelines and frameworks, if any, make a difference. In this paper, we explore potential reuse features through a literature review and present a case study on datasets on GitHub, a popular open platform for sharing code and data. We describe a corpus of more than 1.4 million data files, from over 65,000 repositories. Using GitHub's engagement metrics as proxies for dataset reuse, we relate them to reuse features from the literature and devise an initial model, using deep neural networks, to predict a dataset's reusability. This work demonstrates the practical gap between principles and actionable insights that allow data publishers and tools designers to implement functionalities that provably facilitate reuse.

## Teaser

There is plenty of advice on how to make a dataset easier to reuse, including technical standards, legal frameworks, and guidelines. This paper begins to address the gap between this advice and practice. To do so, a compilation of reuse features from literature is presented. To understand how they look like in data projects, we carried out a case study of datasets published and shared on GitHub, a large online platform to share code and data.

## 1 Introduction

There has been a gradual shift in the last years from viewing datasets as byproducts of (digital) work to critical assets, whose value increases the more they are used.^1^^,^^2^ However, our understanding of how this value emerges, and of the factors that demonstrably affect the reusability of a dataset is still limited.

Using a dataset beyond the context where it originated remains challenging for a variety of socio-technical reasons, which have been discussed in the literature;^3^^,^^4^ the bottom line is that simply making data available, even when complying with existing guidance and best practices, does not mean it can be easily used by others.^5^

At the same time, making data reusable to a diverse audience, in terms of domain, skill sets, and purposes, is an important way to realize its potential value (and recover some of the, sometimes considerable, resources invested in policy and infrastructure support). This is one of the reasons why scientific journals and research-funding organizations are increasingly calling for further data sharing^6^ or why industry bodies, such as the International Data Spaces Association (IDSA) (https://www.internationaldataspaces.org/) are investing in reference architectures to smooth data flows from one business to another.

There is plenty of advice on how to make data easier to reuse, including technical standards, legal frameworks, and guidelines. Much work places focus on machine readability and interoperability.^2^ For example, the Joint Declaration of Data Citation, a statement endorsed by 120 research organizations, affirms that “sound, reproducible scholarship rests upon a foundation of robust, accessible data.”^7^ There is an increasing drive to publish scholarly data in line with FAIR principles, that is to make data Findable, Accessible, Interoperable, and Reusable.^2^ The Share PSI group (https://www.w3.org/2013/share-psi/bp/) made similar recommendations for data published by public administrations. More recently, governments and researchers have started to explore different approaches to data governance, as a way to foster growth and competition in areas heavily disrupted by artificial intelligence, including transport^8^ and finance.^9^

While some of the technologies and guidelines are more widely used than others, most existing work in this space remains normative and lacks operational detail. As a community, we know very little and have even less measurable evidence on what makes a dataset more reusable.

The aim of this paper is then to begin to bridge the gap between such normative guidelines and operational details. We do this through a review of the literature coupled with a deep dive into a specific case study. Concretely, the contributions of the paper are:

1. a compilation of reusability features of datasets and of the mechanisms used to publish and share them, which are commonly linked to reusability, based on a literature review
2. a large corpus of 1.47 million datasets from 65,537 data repositories and their characteristics made available via GitHub, (https://github.com/laurakoesten/Dataset-Reuse-Indicators) a popular platform used to share data science work
3. a case study that uses a five-step approach to understand projected data reuse in a particular corpus context: including a machine learning model to estimate how much a dataset will be reused based on features of the repository where it was published, the actual data, and its documentation, trained on the GitHub corpus

From the literature, we identify features pointing to reusability of datasets that can be captured automatically (or semi-automatically). We then determine correlations between these and actual reuse, using engagement metrics of the platform where the data was published as proxies for reuse.

Several widely shared platforms for sharing and reusing data are available online, such as GitHub—originally focused on code reuse or other more recent ones, such as Kaggle, (https://www.kaggle.com/) data.world, (https://data.world/) or governmental data portals, focusing more on datasets. Each of those are unique, not just in how they are built and how data can be retrieved, but also in the interactions they support and track. We focus this work on GitHub as one of the largest and most widely used collaborative platforms with a large amount of datasets.

We select a set of four GitHub-specific engagement metrics: the number of forks, watchers, stars, and committers. We then create a model that predicts how likely it is for a dataset to be reused on a four-point scale, reaching an accuracy of 59% in the highest reusability category for our corpus.

This case study provides an indication that such an approach can help flag areas of improvement in how datasets are published, for instance, around documentation; as well as monitor the uptake of openly available datasets, by prioritizing those reuse features that are likely to have higher impact on engagement.

The findings confirm a tension between, on the one hand, initiatives promoting data reuse principles and technical standards, and, on the other, operational, automated approaches that allow data publishers and system designers to capture reuse in terms of specific, observable features and provide actionable suggestions for improvement. The findings also point to several under-explored opportunities to encourage and facilitate dataset reuse on the web. We outline a potential direction to further develop both, guidance for dataset reuse as well as functionalities to predict a dataset's reusability. We also recommend missing information to be added at the time of data publishing to enhance the value of existing dataset and enable meaningful reuse by wider audiences.

## 2 Dataset Reuse: the View of the Literature

We summarize guidance and recommendations for dataset reuse from the literature, drawing on several areas, including data science, information science, scientific data sharing, and human-data interaction. We begin by setting the overall context of the value of data through reuse, in particular in the context of FAIR data. We then present a compilation of data reuse features. In the section entitled “GitHub Case Study” we will link these features to platform-specific reuse metrics and present a machine learning model that can predict how much a dataset will be reused.

### 2.1 Why Reuse?

Data reuse has many economic and societal benefits—it facilitates reproducible research, and fosters innovation and collaborations.^10^, ^11^, ^12^ Providing access to the data is a first important step to reap these benefits. Equally important is to make this data easy to use by people who were not involved in its publication.^13^

One of the key challenges to accessibility and uptake of the data published on the web is to create supporting formats and capabilities to make it useful in as many contexts as possible.^14^ Reuse is more common in some domains than in others. For example, scientists reuse data of their peers to reproduce previous experiments; as such the value of data management and documentation to scientific work is increasingly recognized.^4^ Developers define benchmark datasets and gold standards that everyone can use to compare related algorithms and approaches.^15^^,^^16^ They reuse datasets to ensure that approaches remain comparable. Machine learning is dependent on the availability of relevant datasets to train algorithms. In this case, reuse is an economic necessity—machine learning architectures need to be trained on large amounts of data and not many organizations can afford to create them from scratch.^17^

Data are recognized as an asset in itself, cited and archived just like scientific literature.^18^ Policy makers are devising new regulation to ensure access to high-value data assets as a means to promote open science^19^ and make markets more competitive.^9^^,^^20^

### 2.2 Capturing Context and Documentation as Pre-requisites to Reuse

There are many factors that impact on one's ability to reuse data. Documentation and context are commonly recognized as essential.^3^^,^^4^ This had led to efforts to anticipate future uses of data and preserve and describe them. As new use cases arise, the data science community has suggested ways to augment these descriptions—for instance, machine learning specialists need information about the quality and bias in the data to inform model building.^21^^,^^22^ As a result, we now have a range of proposals for standardized documentation for data, going beyond metadata schemas and vocabularies, such as DCAT (https://www.w3.org/TR/vocab-dcat-2/) or schema.org, (https://schema.org/Dataset) which are primarily used to search and browse dataset repositories.^21^^,^^23^^,^^24^

Barriers to, as well as motivations for data sharing have been investigated qualitatively (e.g., in Van den Eynden et al.^25^). People struggle to understand data without context, and while context can mean different things, reuse without any context reference is almost impossible to do well.^26^^,^^27^ When discussing reuse, we also need to take into consideration the social role that data play in producing scientific work.^11^^,^^28^ Similarly, we have to acknowledge the complex decision-making processes that feed into the creation of a dataset.

Some authors argue that each domain (and ultimately each type of data) will present its own requirements for reuse.^29^ We delve into the difference between quantitative versus qualitative data reuse in more detail below. However, even with such disciplinary distinctions, our paper shows one way to improve current reuse practices, before focusing on domain-specific requirements.^25^

### 2.3 Different Practices for Quantitative and Qualitative Data

Different data science methodologies may require different ways to document data. Broadly speaking, it is more common to reuse quantitative data than qualitative data.^30^ Carlson and Anderson^31^ comment on how highly individualized data collection processes are across quantitative and qualitative disciplines. The nature of null-hypothesis significance testing, which is common in the former, leads to efforts to reduce confounding factors as much as possible, hence creating a data environment with clear cut boundaries, which should, in theory, be easy to document.^32^ In reality, many authors describe the complexities and variety of decision-making points in this type of analysis, which pose challenges for reuse.^33^^,^^34^ Nevertheless, a detailed account of the experimental set-p is common for quantitative methods; the same would be beneficial to support the reuse of qualitative data, which, some authors claim, is more situated within its context and hence presents more barriers to reuse or secondary use.^35^^,^^36^

Reuse of qualitative data comes with unique challenges, many of them connected to ethical considerations, as explained in detail by Poth.^35^ One of the main issues is that original consent forms often do not include the possibility of data reuse, or are not available to the data consumer. Archives of social sciences data, such as the UK data archive, now integrated with the UK Data Service (https://ukdataservice.ac.uk/) or GESIS (www.gesis.org/) in Germany point toward the existing practice of qualitative data reuse.

In Koesten et al.,^37^ the authors describe the information structures needed for both qualitative and quantitative data reuse among researchers, highlighting that, while there is a lot of overlap, there are also certain aspects of the study design worth detailing for specific methods, due to their high impact on results. This includes, for instance, information about whether a survey question is required or not, because, for the former, participants are more likely to select a random answer to be able to continue, also mentioned by Koesten et al.^37^

### 2.4 Existing Guidance and Principles and Their Limitations

As noted in Section 1, the FAIR principles are a strong example of community and policy push toward more reusable data.^38^ Thus, they provide an important reference point for thinking about data reuse.

The FAIR principles are a compilation of high-level, trans-disciplinary best practices for making data findable, accessible, interoperable, and reusable.^2^ One of the key messages in FAIR data science is that metadata and metadata standards should be articulated and made publicly available to the greatest extent possible.^39^ The “R” in FAIR is about reusability and refers to the following points, focusing primarily on metadata:

1. meta(data) should be richly described, with a plurality of accurate and relevant attributes
2. (meta)data should be released with a clear and accessible data usage license
3. (meta)data should link to detailed provenance information
4. (meta)data should meet domain-relevant community standards

There are a variety of other proposals for data publishing, sharing, and reuse, which follow similar aims. Some of them focus on a sector (e.g., SharePSI (https://www.w3.org/2013/share-psi/) for public administration) or on a set of technologies (e.g., the web mark-up vocabularies, such as Dublin Core, (https://www.dublincore.org/groups/tools/) DCAT, (https://www.w3.org/TR/vocab-dcat/) schema.org,https://schema.org/Dataset and PROV (https://www.w3.org/2001/sw/wiki/PROV)) or on data quality (e.g., W3C (https://www.w3.org/TR/vocab-dqv/)). Implementing these standards to a sufficient quality level to enable data reuse is often difficult.^40^, ^41^, ^42^

Measuring “FAIRness” is not yet an established practice,^43^ although some initial work exists, thanks to the FAIR metrics group (http://fairmetrics.org). This means, among other things, that someone making the effort into publishing their data according to (their interpretation of) FAIR, has limited ways to gauge how meaningful their work is in practice. An uptake in data citation may very well help with this, although to date we have not seen a comprehensive overview of actionable, general-purpose reuse indicators.

Domain-specific efforts, such as the Minimum Information Standards or Models in the Life Sciences have emerged as a means of more standardized reporting of experiments to increase the quality and reusability of data.^44^ These standards or models are a collection of domain-specific guidelines and checklists. They originated in the Biological and Biomedical domain but are being extended to other areas (https://fairsharing.org/). These standards target a similar problem that we aim to address in this work, namely: narrowing down documentation effort to the minimum required for others to reuse data. In the case of these Minimum Information Standards, there a focus on community-defined guidelines for experimental data in a domain. In contrast our work can be seen as a higher-level approach, focusing on general-purpose scenarios that can be operationalized broadly.

### 2.5 Reuse Features

Having introduced reuse from different angles, we now focus on compiling a list of reuse features (Table 1). This list is informed by a review of the literature covering works from several disciplines, which consists of 40 papers published from 1996 to 2019. For each paper, we looked for principles and guidance around processes and technologies for data sharing and reuse practices. Some of the resulting features are mentioned in relation to specific domains—in those cases, we kept only those that we came across in several papers from different communities, or those we thought to be more widely applicable.

**Table 1:** Compilation of Reusability Features for Datasets

Feature	Description	References
**Access**		

License	(1) available, (2) allows reuse	W3C https://github.com/laurakoesten/^3^^,^^22^^,^^45^, ^46^, ^47^
Format/machine readability	(1) consistent format, (2) single value type per column, (3) human as well as machine readable and non-proprietary format, (4) different formats available	W3C^2^^,^^22^^,^^48^, ^49^, ^50^
Code available	for cleaning, analysis, visualizations	51, 52, 53
Unique identifier	PID for the dataset/ID's within the dataset	W3C^2^^,^^53^
Download link/API	(1) available, (2) functioning	W3C^47^^,^^50^

**Documentation: Summary Representations and Understandability**		

Description/README file	meaningful textual description (can also include text, code, images)	^22^^,^^54^^,^^55^
Purpose	purpose of data collection, context of creation	^3^^,^^21^^,^^49^^,^^56^^,^^57^
Summarizing statistics	(1) on dataset level, (2) on column level	^22^^,^^49^
Visual representations	statistical properties of the dataset	^22^^,^^58^
Headers understandable	(1) column-level documentation (e.g., abbreviations explained), (2) variable types, (3) how derived (e.g., categorization, such as labels or codes)	^22^^,^^59^^,^^60^
Geographical scope	(1) defined, (2) level of granularity	^45^^,^^54^^,^^61^^,^^62^
Temporal scope	(1) defined, (2) level of granularity	^45^^,^^54^^,^^61^^,^^62^
Time of data collection	(1) when collected, (2) what time span	63, 64, 65

**Documentation: Methodological Choices**		

Methodology	description of experimental setup (sampling, tools, etc.), link to publication or project	^3^^,^^13^^,^^54^^,^^60^^,^^63^^,^^66^
Units and reference systems	(1) defined, (2) consistently used	^54^^,^^67^
Representativeness/Population	in relation to a total population	^21^^,^^60^
Caveats	changes: classification/seasonal or special event/sample size/coverage/rounding	^48^^,^^54^
Cleaning/pre-processing	(1) cleaning choices described, (2) are the raw data available?	^3^^,^^13^^,^^21^^,^^68^
Biases/limitations	different types of bias (i.e., sampling bias)	^21^^,^^49^^,^^69^
Data management	(1) mode of storage, (2) duration of storage	^3^^,^^70^^,^^71^

**Documentation: Quality**		

Missing values/null values	(1) defined what they mean, (2) ratio of empty cells	W3C^22^^,^^48^^,^^49^^,^^59^^,^^60^
Margin of error/reliability/quality control procedures	(1) confidence intervals, (2) estimates versus actual measurements	^54^^,^^65^
Formatting	(1) consistent data type per column, (2) consistent date format	W3C^41^^,^^65^
Outliers	are there data points that differ significantly from the rest	^22^
Possible options/constraints on a variable	(1) value type, (2) if data contains an “other” category	W3C^72^
Last update	information about data maintenance if applicable	^21^^,^^62^
Completeness of metadata	empty fields in the applied metadata structure?	^41^
Abbreviations/acronyms/codes	defined	^49^^,^^54^

**Connections**		

Relationships between variables defined	(1) explained in documentation, (2) formulae	^21^^,^^22^
Cite sources	(1) links or citation, (2) indication of link quality	^21^
Links to dataset being used elsewhere	i.e., in publications, community-led projects	^21^^,^^59^
Contact	person or organization, mode of contact specified	W3C^41^^,^^73^

**Provenance and Versioning**		

Publisher/producer/repository	(1) authoritativeness of source, (2) funding mechanisms/other interests that influenced data collection specified	^21^^,^^49^^,^^54^^,^^59^^,^^74^^,^^75^
Version indicator	version or modification of dataset documented	W3C^50^^,^^66^^,^^76^
Version history	workflow provenance	W3C^50^^,^^76^
Prior reuse/advice on data reuse	(1) example projects, (2) access to discussions	^3^^,^^27^^,^^59^^,^^60^

**Ethics**		

Ethical considerations, personal data	(1) data related to individually identifiable people, (2) if applicable, was consent given	^21^^,^^57^^,^^71^^,^^75^

**Semantics**		

Schema/Syntax/Data Model	defined	W3C^47^^,^^67^
Use of existing taxonomies/vocabularies	(1) documented, (2) link	W3C^2^

This table does not claim to be comprehensive but aims to provide an overview of the many recommended documentation practices for dataset reuse. W3C refers to The Data on The Web Best Practices Vocabulary (https://www.w3.org/TR/vocab-dqv/)

We grouped the features into eight categories, all related to the context in which a dataset was created and meant to be used and the related documentation. These categories are: (1) access; (2) summaries and understandability; (3) methodological choices; (4) data quality; (5) connections; (6) versioning and provenance; (7) ethics; and (8) semantics.

#### 2.5.1 Access

Access to data is among the most commonly mentioned attributes in the reuse literature. This includes the display of a dataset’s license and format, which are established practice on data publishing platforms. Restrictive and missing licenses create downstream reuse implications.^46^ A clear access mechanism, such as a download link or API encourages reuse and its operationalization is paramount for reusability. Authors have also included the availability and executability of the code that was used to generate data, which is increasingly requested by scientific journals (https://www.nature.com/sdata/policies/editorial-and-publishing-policies/#code-avail).

Recent approaches develop means to access remote data by allowing differentiated access to retrieve or run code remotely, without gaining access to the raw data.^77^ This facilitates the analysis of potentially sensitive data without physically sharing the data, in a protected environment. While our work does not cover this scenario directly, we assume most of the reuse features are equally applicable, independently of whether the data can be accessed directly. Essentially, many of the attributes necessary to reuse data are independent of having direct access to the data.

#### 2.5.2 Summaries and Understandability

The process of data collection, processing, and cleaning that takes place before a dataset is published can be very complex and is often not reflected in the dataset itself, nor in the documentation attached to it.^56^

Summarizing elements can include text, such as in the description of a dataset,^62^^,^^78^ visual summaries of statistics or trends represented in the data,^79^ or more sophisticated statistical representations.^22^

Marchionini and White^80^ distinguish between overviews, or “surrogates,” and metadata: the former are designed to support people to make sense of the information object before fully engaging with the object itself, whereas the latter are mostly for machine consumption and filtering. Many data publishing platforms include a file that serves as context for the dataset, often describing its purpose. For instance, README files on GitHub can contain a variety of formats, such as text, images, code, or tables.

Increasingly, there is a trend to display column-level summaries (e.g., on Kaggle (https://www.kaggle.com/)), indicating descriptive statistics, units of measurements, expected value types linked to a schema, and value constraints, pointing to methodological choices. Some of these are also included in existing standards and recommendations, for instance, by the W3C, to make it easier for people to make sense of the data.^45^

Related to this category is the understandability of headers. This includes a definition, or if needed a narrative, of how categories used in the data were created or derived from the data.^22^^,^^59^^,^^60^

Visual representations displaying statistical properties of the dataset or analysis results are mentioned in the literature.^22^^,^^58^ Similar to describing the datasets methodology, the more transparent choices and processes of these visual representations are made, the easier it is for a user to understand their value.

The importance of spatial and temporal boundaries for data reuse is recognized in the literature and practitioners' guides alike.^45^^,^^61^^,^^81^^,^^82^ Representations of granularity allow data consumers not just to judge whether the data cover the desired location and dates, but also whether the level of aggregation makes it suitable for the task at hand. Related to the temporal scope of a dataset, but a more structural indicator, are indications of the time of data collection as well as the last update or expected frequency of updates and maintenance of the dataset (which is mentioned as an aspect of data quality in Table 1).

#### 2.5.3 Methodological Choices

There is general consensus that information about the methodological basis on which a dataset was created is necessary for informed reuse. However, the concept of methodology remains vague in many recommendations or is tied to a particular domain. We describe a general-purpose view on common denominators of choices during dataset creation that are said to be necessary for dataset reuse.

The importance and difficulties of understanding a dataset's context of creation and the decisions taken by those compiling and organizing the data is mentioned frequently.^3^^,^^48^^,^^60^^,^^83^ We focus on those aspects that could be expressed in actionable indicators, rather than a wider discussion of context, common in the reuse literature. A recent example of this broad focus on context is in Faniel et al.,^3^ in which context includes a wide range of methodological characteristics, as well as the producer and data analysis.

Methodological choices include detailed accounts of every aspect of the experimental design, including its setup, testing, and cleaning of the data. The level of detail depends on the type of data and the type of reuse and will hence have to be decided for each dataset to strike a balance between publisher effort and likely reuse scenarios.

Aside from describing the creation strategy of the dataset, this can also include units and reference systems used in the data,^54^^,^^67^ cleaning and pre-processing protocols,^3^^,^^13^^,^^21^^,^^68^ and pointers to other information sources, such as code. For instance, Carlson and Anderson^31^ mention the algorithm used to calibrate the device for an experiment.

Documenting methodology also includes potential biases and limitations due to choices in the datasets creation. Kale et al.^33^ discuss how researchers convey uncertainties, such as the assumptions and constraints behind their analysis by writing caveats in limitations sections or preparing supplemental presentation slides. Equally, information about data management strategies, including how data are stored and preserved on a particular type of storage medium, help paint a more complete picture of a dataset, and can be critical to automate processing.^3^^,^^70^^,^^71^

#### 2.5.4 Data Quality

Data on the web contain inconsistencies, and incomplete and misrepresented information. At the same time, quality is not a fixed characteristic of a dataset, but depends on the task. Data quality is commonly described as “fitness for use” for a certain application or use case.^65^^,^^84^ Quality assessment may depend on various factors (dimensions or characteristics), such as accuracy, timeliness, completeness, relevancy, objectivity, believability, understandability, consistency, conciseness, availability, and verifiability.^65^ Koesten et al.^62^ collected perceptions of data quality in the context of dataset selection, including provenance or descriptions of methodology, which we discuss separately. Quality has been studied in relation to specific data formats. For instance, Zaveri et al.^85^ analyzed quality dimensions focusing on linked data, a set of technologies recommended to publish data on the web to aid interoperability across applications.^86^ They defined four core dimensions: accuracy, completeness, consistency, and timeliness.

Despite the efforts, data quality dimensions are not easily transferable across domains.^87^ However, a number of quality metrics for structured data have been proposed in the literature, such as metrics for correctness of facts, adequacy of semantic representation, and the degree of coverage.^72^^,^^85^ Consistent formatting and the machine readability of a dataset (as mentioned under access) have also been stated as quality indicators.^2^^,^^50^

Discussions of data quality often include how well an awareness of uncertainty attached to the dataset is communicated,^33^^,^^37^ as well as the negotiation of potential biases or the meaning of missing values. All these aspects often require the user to access additional information, and remain challenging.^69^ This indicates that data quality is inseparable from documentation efforts, be that as metadata or other forms of contextual material.

Above all, quality is task dependent, hence aspects, such as missing values or categorization procedures can determine quality perception.^88^ For instance, certain tasks are more sensitive to missing data than others, which means information about missing data can be crucial to evaluate fitness for use (e.g., Koesten et al.^5^). Missing data has been discussed in depth from a statistical point of view, including different methods to tackle it (e.g., Little^89^). Not many studies have looked at interaction challenges in dataset reuse resulting from missing data. Missing data can mean different things and the meaning should be documented to facilitate understanding.

##### 2.5.4.1 Metadata Quality

Other authors discuss the quality of metadata, rather than the data to be a defining factor in assessing open data quality. Umbrich et al.^41^ point out that low metadata quality (or missing metadata) affects both the discovery and the consumption of the datasets. In that sense, the quality of metadata can be seen as one aspect of data quality, but includes in itself a number of different concepts (such as completeness, accuracy, or openness; among others^41^).

#### 2.5.5 Connections

We refer here both to connections within a dataset as well as to connections to external resources. Within a dataset this includes relationships between variables through formulae or other dependencies that state relations within the data.^21^^,^^22^ For instance, several columns referring to a location but in different levels of granularity, or a column being the result of a calculation between other columns. Connections outside the data may include links to sources of the data,^21^ to the dataset being used elsewhere (e.g., links to projects or dataset citations), as well as contact information for the authors or owners of the data.^59^^,^^73^ These may or may not be directly actionable (i.e., working links).

#### 2.5.6 Versioning and Provenance

Versioning information is often linked to reusability.^66^^,^^90^ Version numbers make a revision of a dataset uniquely identifiable.^45^ Similarly to code, datasets evolve over time. Version histories help track choices in the curation of a dataset and revert to the most suitable version, facilitating reuse.^50^

There are different definitions of provenance in the literature: narrower ones refer to the data producer and the publishing institution, (https://dublincore.org/) wider ones include a broad description of datasets or data points lineage,^91^^,^^92^ overlapping with what we discussed under methodological choices. Information about the publisher can give an indication of the authoritativeness of the source, but should also inform about the funding mechanisms or potential other interests that could have influenced data collection practices (e.g., Faniel and Yakel^59^). Some disciplines have their own reference datasets offered by authoritative sources.^93^

Provenance information has been discussed widely in literature (e.g., Herschel et al.^92^ and Moreau and Groth^94^ ) and can give an indication of the authoritativeness, trustworthiness, context, and purpose of a dataset to make sense of it and assess its integrity and value. This includes information on publisher and/or data producer as well as a contact point for questions or community engagement around the dataset.^45^^,^^95^

Provenance is sometimes understood as a dataset's traceability.^65^^,^^66^ It has conceptually found application in provenance trails to automatically create application-level provenance information during workflows.^96^^,^^97^

Similarly, information about prior reuse, as well as advice on data reuse are said to support reusability. This is an emerging practice across data science communities, as it can be seen for instance, on Kaggle, (https://www.kaggle.com/) where datasets are discussed via example projects.^3^^,^^59^

#### 2.5.7 Ethics

Ethical considerations and the documentation or protection of personal data are a complex and multi-layered category in themselves. Our aim here is to provide a general-purpose perspective with a focus on reuse, rather than a comprehensive introduction into frameworks and techniques, such as for anonymization. This includes considerations of identifiability, especially if combined with other data sources and questions of consent, laws and regulations, and ethical-review processes, but also whether the data collected represents social groups fairly or whether it contains potentially sensitive content. This is discussed in more detail by authors, such as Gebru et al.,^21^ Holub et al.,^75^ and Knoppers.^71^

##### 2.5.8 Semantics: Taxonomies, Schemas, and Vocabularies

Data are encoded in a specific way, using technical schemas, data models, and language dialects. Using existing vocabularies and other knowledge structures to organize a dataset enhances its understandability;^2^^,^^45^^,^^67^ the meaning of attributes is documented in the vocabulary and, as more and more datasets use the same structure, they become easier to integrate. In this context, it is also important to note the importance of vocabulary extensions, to fit specific use cases while still maintaining a common core.

To summarize this section, we found a large number of potential reuse requirements, guidelines, and recommendations in the literature. However, in most cases their definition leaves room for interpretation or they are implemented in various ways across data publishing and sharing platforms. Our aim is to go a step further, taking Table 1 as a starting point to develop more quantifiable measures, which can be provably linked to reuse.

## 3 GitHub Case Study

Data achieve their impact if they are widely reused. Our literature review has produced a comprehensive list of features of datasets and related processes, which should be considered by data producers and system designers to make their data easier to use by others. In this section, we use a case study to explore an approach that grounds these activities into actionable steps and metrics. By understanding which aspects of dataset publishing and use impact reusability, one could potentially improve publishing practice, iterate over the design of portals and other sharing platforms, and prioritize publishing and maintenance work.

We organize our case study adopting a five-step approach that we formulate as high-level steps to suggest the potential of applying the concept to other data reuse contexts in future work:

1 Corpus building—scope the assessment exercise, for instance, by deciding the specific collection of datasets that will be considered.
2 Features and metrics—define reuse features and metrics and ways to measure them. For the features, consider those from Table 3 as a starting point. If you do not have a standard data reuse metric, think about proxy metrics and validate them. Both features and metrics will depend on the capabilities of the data publishing medium and the underlying technical infrastructure.
3 Data collection and analysis—for each feature, you will need to decide how you will measure it. Some features will be straightforward, like establishing whether a link is available or not. Others will require custom techniques. For the metrics, you can rely on technical capabilities, which may be built into the publishing software you are using, or compile aggregated metrics derived from lower-level system logs.
4 Reuse prediction—build a statistical model to predict reusability, informed by the analysis from the previous step. Train and test the model.
5 Recommendations—take action and derive recommendations to datasets, processes and system capabilities.

While engagement metrics and the interactions captured will vary for each portal and dataset corpus, we believe there is value in presenting this approach as a potential direction of conceptualizing and advancing efforts to increase dataset reuse. The machine learning model, detailed later, follows a modular design to simplify its adaption in other dataset-reuse-prediction tasks. Hence, this approach could potentially be adopted by data publishers, repository managers, or system designers. Our model considers each potential type of reuse equally. Hypothetically different types of reuse could be modeled differently, depending on the complexity of the scenario.

For our case study, we downloaded a large corpus of datasets from GitHub, a popular platform for sharing code as well as datasets with an accessible, extensive, and varied collection of structured data. First, we used descriptive statistics to understand the engagement patterns and thus potential reuse and took a qualitative look at the highly reused repositories to understand their documentation practices. Secondly, we built a predictive model to attempt to link indicators as derived from the literature to these engagement proxies. We now describe each of these steps but first begin with a description of how we constructed the corpus. The annotated corpus and the code are available on GitHub (https://github.com/laurakoesten/Dataset-Reuse-Indicators).

## 4 Corpus Building

For the purpose of our analysis, we used the following working definition of a data repository on GitHub: a repository that has tabular data of a minimum size of ten rows in a CSV, XLSX, or XLS file type.

We used Google's public dataset copy of GitHub and the BigQuery service (https://cloud.google.com/bigquery/public-data) to build an original list of repositories (that were not forks of existing repositories) that contain a CSV or XLSX or XLS file. We then used the GitHub API to collect information about each repository in this original list.

The resulting dataset consists of 87,936 repositories that contain at least a CSV, XLSX, or XLS file, alongside complementary information on their features (e.g., number of open and closed issues and license) from GitHub. This corpus had more than two million data files. We then excluded those files with less then 10 rows, which was the case for 65,537 repositories with a total of 1,467,240 data files. From these, 1,373,335 were CSV files, 56,485 were XLSX files, and 37,420 were XLS files. Per repository, we found an average of 7.4% ± 13.4% data files (med: 1.852%). With very few exceptions all repositories have an associated license.

Table 2 summarizes the statistics for the entire dataset corpus. The top languages as provided by the GitHub API can be seen in Table 7.

**Table 2:** Characteristics of the Dataset Repository Corpus Used in This Study

Type	Characteristics	Mean (±SD)	Quantile
Data file	no. of rows (csv)	4,115 (±50,094)	[39.0, 92.0, 108.0]
	no. of columns (csv)	20.5 (±373)	[3.0, 5.0, 12.0]
	no. of rows (xls(x))	607 (±13610)	[28.0, 65.0, 108.0]
	no. of columns (xls(x))	30.5 (±412.1)	[8.0, 15.0, 19.0]
	no. of missing values csv (ratio)	8.9 (±17.5)	[0.0, 0.0, 11.5]
	avgerage size of data files (csv)	331,343 (±3,719,328)	[1,625.0, 8,375.0, 47,752.5]
	average size of data files (xlsx)	428,586(±2,595,222)	[18,804.0, 34,723.0, 121,633.0]
Repository	size of repository	51,372 kilobytes (±211,729)	[983.0, 7,740.0, 32,715.0]
	no. of open issues	5.2 (±51.2)	[0.0, 0.0, 0.0]
	no. of closed issues	40.6 (±552.3)	[0.0, 0.0, 2.0]
	description length	7.2 (±9.2)	[1.0, 5.0, 10.0]
	ratio of data files per repo	7.2% (±13%)	[0.3, 1.9, 8.0]
	age of repository (days)	1,521.9 (±539.7)	[1,108.0, 1,478.0, 1,844.0]
	ratio of problematic files with respect to a standard config (Pandas)	0.3% (±2.6%)	[0.0, 0.0, 0.0]
README	no. of words in README (non-code related)	378.2% (±1,126.6%)	[10.0, 112.0, 431.0]
	no. of tables	0.1 (±1.0)	[0.0, 0.0, 0.0]
	no. of code blocks	1.4 (±4.7)	[0.0, 0.0, 1.0]
	no. of headers	3.6 (±17.0)	[1.0, 1.0, 5.0]
	no. of urls	9.1 (±36.9)	[1.0, 3.0, 12.0]
	no. of images	0.7 (±3.9)	[0.0, 0.0, 0.0]

Average values are reported in the “mean (±SD)” format. Quantiles values are reported in the [$x_{25}$, $x_{50}$, $x_{75}$] format, where $x_{25}$, $x_{50}$ and $x_{75}$ represent the 25th, 50th, and 75th quantile of a particular group's characteristic.

## 5 Reuse Metrics and Features

### 5.1 Reuse Metrics

In our case study, we identified a set of engagement metrics with datasets published on GitHub that are indicative of reuse and available via the GitHub API.

Watchers: watching a repository registers the user to receive notifications on new discussions, as well as events in the user's activity feed.

Forks: creating a fork describes producing a personal copy of someone else's project.

Committers: number of parties, identified via email addresses, which have committed on the master branch. Note that it is possible that the same person commits with different email addresses.

Stars: repository starring lets users bookmark repositories. Stars are shown next to repositories to show an approximate level of interest and have no effect on notifications or the activity feed.^24^

### 5.2 Reuse Features

We mapped the features presented in Table 1 to GitHub. We considered three sources of data to populate these features: (1) the repository where the dataset was published; (2) the README file as the main documentation of the work; and (3) the data files themselves. Like our engagement metrics, the features we use are only proxies, but provide a useful and importantly measurable starting point for more standardized indicators of dataset reuse (Table 3).

**Table 3:** Features Used as Proxies for Reuse in the GitHub Case Study

Repository	README File	Data Files
(I) age of repository (in days) $r^{\left(a\right)}\;\in N$	(I) length of the README (no. of tokens) $r^{\left(g\right)}\;\in N$	(I) no. of ROWS of each individual data file $f^{\left(r\right)}\;\in N$
(II) size of repository (in kb) $r^{\left(s\right)}\;\in N$	(II) unique URLs $t^{\left(u\right)}\;\in N$	(II) no. of COLUMNS of each individual data file: $f^{\left(c\right)}\;\in N\;f^{\left(c\right)}$
(III) license of repository $r^{\left(I\right)}$ represented as a one-hot input vector whose dimensionality equal to the total number of different licenses in the dataset	(III) language of the README (English or not): $t^{\left(u\right)}\;\in \;\left\{0,1\right\}$	(III) missing values (ratio of missing values to total values): $f^{\left(n\right)}\;\in \;\left[0,1\right]$
(IV) textual description $r^{\left(x\right)}\;=x_{1},x_{2},\ldots \;x_{T}\;\text{Where}\;x_{1},x_{2},\ldots x_{T}$is the sequence of words from which a data repository's description consists	*(*IV) no. of inline coding blocks: $t^{\left(b\right)}\;\in N$	(IV) size of each data file (kb): $f^{\left(s\right)}\;\in N$
(V) ratio of open to closed issues: $r^{\left(i\right)}\;\in \;\left[0,1\right]$	(V) no. of highlighting coding blocks: $t^{\left(f\right)}\;\in N$	–
(VI) ratio of data files to all files in a repository: $r^{\left(f\right)}\;\in \;\left[0,1\right]$	(VI) no. of headers: $t^{\left(h\right)}\;\in N$	–
(VII) ratio of problematic files with respect to a standard configuration: $r^{\left(n\right)}\;\in \;\left[0,1\right]$	(VII) no. of tables: $t^{\left(t\right)}\;\in N$	–
	(VIII) no. of images: $t^{\left(i\right)}\;\in N$	–

As noted earlier, these features will feed into the model that predicts reusability, as explained in Sections 6 and 7.

## 6 Data Collection and Analysis

### 6.1 Reuse Metrics

#### 6.1.1 Data Collection

Engagement data were collected via the GitHub API as follows:

Watchers: watchers are called subscribers in the GitHub API.^25^ We collected watcher count by calling the API iteratively.^26^

Forks: similar to the case of watchers, we collected forks count by calling the API iteratively.^26^

Committers: as noted earlier, we considered number of different email addresses that have committed on the master branch. We collected these counts by using regular expressions on each data repository.git file.

Stars: repository starring lets users bookmark repositories. Stars are shown next to repositories to show an approximate level of interest and have no effect on notifications or the activity feed.^27^

#### 6.1.2 Data Analysis

##### 6.1.2.1 Descriptive Statistics and Correlation Analysis

Table 4 summarizes the basic data for the four engagement metrics in our corpus.

**Table 4:** Engagement Metrics: Proxies for Reuse

Metrics	Mean (±SD)	Median
No. of watchers (subscribers)	6.1 (±50.1)	0…1.0…8581
No. of forks	15 (±378.8)	0; (max 77,118)
No. of committers	28.3 (±604)	2; (max 24,463)
No. of stars	40.8 (±797.4)	0; (max 133,515)

We note that stars and watchers show the highest correlation, which might be due to them being treated similarly in the interface (ρ=0.0.57, p <0.001). There is also a high correlation of forks and stars (Spearman ρ=0.69, p <0.001)/and with watchers (ρ=0.57, p <0.001). Forks have a lower correlation with committers (Spearman ρ=0.38, p <0.001). Stars and watchers show a high correlation (ρ=0.57, p <0.001), this might be because they are treated similarly in the interface. Committers are highly correlated with watchers (ρ=0.46, p <0.001). Commits are different, they do not correlate linearly. The top repositories have around 25,000 commits.

##### 6.1.2.2 Grouping and Ranking Repositories by Engagement

To have a clearer picture of those features which are characteristic of increased reuse, we grouped and ranked repositories by engagement. To do so, and to tackle tie scores with respect to the engagement metrics, we opted for a modified version of the Borda count that rewards repositories that have the same engagement counts per metric. This count is also used when the number of elements in a list is large (>30,000) and is popular due to its limited time complexity.^98^ The average number of ties for repositories with a Borda count of over 50 is low (7,025 repositories), which counterbalances the integration of ties using Borda.^98^

We used the aggregated Borda count as a reference to create four reuse “profiles.” Group 1 includes the repositories with the lowest Borda count up to 8, which reflects a minimum of engagement with the repository. Group 2 included those with up to three engagement counts in each category (Borda counts 9–20), group 3 includes those with up to 9 more counts in each category (Borda counts 21–64) and group 4 includes all repositories with more engagement counts (Borda count 65–2,608). Other considerations in group definition were to keep the sample roughly balanced as well as incorporating the distribution of the aggregated ranked list.

• Group 1: 4–8; up to one count, 35,096 repositories
• Group 2: 9–20; up to three more in each category, 16,494 repositories
• Group 3: 21–64; up to nine more in each category, 8,196 repositories
• Group 4: 65–2,608; more than nine in each category, 5,751 repositories

**Figure 1:**
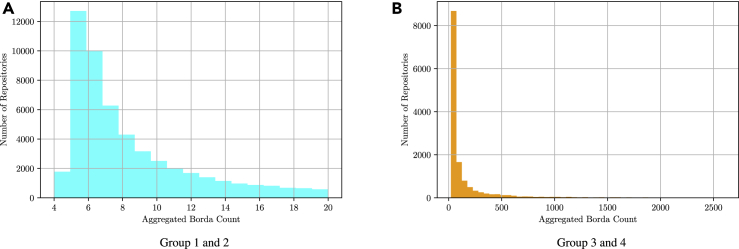
Distribution of Data Repositories according to the Borda Count (A and B) (A) depicts the repositories that belong to groups 1 and 2, and (B) the ones that belong to groups 3 and 4, with respective Borda counts of lower and greater or equal than 21.

Figure 1 shows how data repositories are distributed according to their rank after aggregation.

Table 5 displays average population statistics of the data reuse metrics across the four groups of reuse. Table 6 shows characteristics (mean, standard deviation, and median) of the dataset corpus according to the four groups of reuse. Median values are reported in the $x_{\text{min}}\ldots \tilde{x}\ldots x_{\text{max}}$ format, where $x_{\text{min}}x_{\text{min}}$ is the minimum and $x_{\text{max}}$ the maximum of the *x* variable.

**Table 5:** Characteristics of the Reuse Metrics for Each Group of Reuse: 1 = Lowest Reuse, 4 = Highest Reuse

Characteristics	Mean G1	Mean G2	Mean G3	Mean G4	Quantile G1	Quantile G2	Quantile G3	Quantile G4
Watchers (subscribers)	1.0 (±0.6)	2.8 (±2.1)	7.3 (±6.8)	44.7 (±163.7)	[1.0, 1.0, 1.0]	[1.0, 2.0, 4.0]	[3.0, 5.0, 9.0]	[8.0, 17.0, 37.5]
Forks	0.1 (±0.4)	1.3 (±1.6)	5.5 (±5.8)	158.8 (±1,269.8)	[0.0, 0.0, 0.0]	[0.0, 1.0, 2.0]	[1.0, 4.0, 8.0]	[11.0, 29.0, 79.0]
Committers	1.6 (±0.8)	3.7 (±2.5)	10.2 (±11.4)	287.6 (±2,020.8)	[1.0, 1.0, 2.0]	[2.0, 3.0, 5.0]	[3.0, 6.0, 13.0]	[5.0, 18.0, 62.0]
Stars	0.2 (± 0.5)	1.9 (±2.2)	9.2 (±9.1)	445.1 (±2,658.5)	[0.0, 0.0, 0.0]	[0.0, 1.0, 3.0]	[1.0, 7.0, 14.0]	[19.0, 61.0, 186.0]

**Table 6:** Characteristics of the Dataset Corpus and for Four Groups of Reuse: 1 = Lowest Reuse, 4 = Highest Reuse

Type	Characteristics	Mean G1	Mean G2	Mean G3	Mean G4	Quantile G1	Quantile G2	Quantile G3	Quantile G4
README	no. of words in README (non-code related)^a^	286.2 (± 963.8)	345.1 (±835.6)	541.9 (±1,509.7)	801.9 (±1,808.7)	[6.0, 48.0, 287.0]	[15.0, 125.0, 389.8]	[63.0, 250.0, 626.0]	[151.5, 416.0, 869.0]
	no. of tables^a^	0.0 (±0.5)	0.1 (± 0.6)	0.1 (±1.6)	0.3 (±2.2)	[0.0, 0.0, 0.0]	[0.0, 0.0, 0.0]	[0.0, 0.0, 0.0]	[0.0, 0.0, 0.0]
	no. of code blocks^a^	0.9 (±3.5)	1.3 (±4.2)	2.3 (±6.1)	3.5 (±8.1)	[0.0, 0.0, 1.0]	[0.0, 0.0, 1.0]	[0.0, 0.0, 2.0]	[0.0, 1.0, 4.0]
	no. of headers^a^	2.3 (± 4.1)	3.6 (±5.6)	5.3(±7.9)	8.8 (±54.6)	[0.0, 1.0, 3.0]	[1.0, 1.0, 5.0]	[1.0, 3.0, 7.0]	[2.0, 6.0, 10.0]
	no. of URLS^a^	6.0 (±10.4)	8.1 (±18.4)	12.8 (±21.1)	25.2 (±113.7)	[1.0, 2.0, 8.0]	[1.0, 4.0, 11.0]	[2.0, 8.0, 17.0]	[6.0, 15.0, 28.0]
	no. of images^a^	0.3 (±1.7)	0.7 (±5.5)	1.1 (±4.8)	2.5 (±6.1)	[0.0, 0.0, 0.0]	[0.0, 0.0, 0.0]	[0.0, 0.0, 1.0]	[0.0, 1.0, 3.0]
Repository	repository size^a^	33,689.8 (± 152,529)	50,916.3 (± 194,154)	70,511.1 (±225,835)	133,307.1 (±423,076)	[580.0, 5,386.5, 22,780.2]	[1,230.0, 7,667.0, 33,723.8]	[2,174.5, 14,557.0, 52,912.2]	[4,896.5, 27,393.0, 113,130.0]
	no. of open issues^a^	1.1 (±10.8)	2.0 (±13.2)	6.4 (±21.8)	38.1 (±163.7)	[0.0, 0.0, 0.0]	[0.0, 0.0, 1.0]	[0.0, 1.0, 4.0]	[0.0, 5.0, 25.0]
	no. of closed issues^a^	1.9 (±13.5)	7.6 (±31.7)	38.4 (±130.8)	3,74.7 (±1,823.4)	[0.0, 0.0, 0.0]	[0.0, 0.0, 3.0]	[0.0, 2.0, 19.0]	[2.0, 25.0, 175.5]
	description length^a^	6.2 (± 8.3)	7.7 (±9.2)	8.9 (±11.2)	9.6 (±10.2)	[0.0, 4.0, 9.0]	[2.0, 6.0, 11.0]	[4.0, 7.0, 11.0]	[4.0, 7.0, 12.0]
	ratio of data files per repository^a^	8.2 (±14.0)	7.1 (±12.7)	5.4 (±10.9)	3.6 (±8.7)	[0.2, 2.3, 10.0]	[0.4, 2.2, 7.7]	[0.3, 1.4, 5.3]	[0.1, 0.7, 2.8]
	age of repository (days)^a^	1,467.9 (±490.0)	1,513.4 (±545.2)	1,627.7 (±592.3)	1,725.3 (±653.0)	[1,067.0, 1,448.0, 1,791.0]	[1,093.2, 1,453.0, 1,816.0]	[1,214.0, 1,562.0, 1,964.0]	[1,256.5, 1,628.0, 2,082.5]
	ratio of problematic files for a standard config (Pandas)^b^	0.3 (±2.7)	0.4 (±2.8)	0.3 (±2.6)	0.2 (±1.5)	[0.0, 0.0, 0.0]	[0.0, 0.0, 0.0]	[0.0, 0.0, 0.0]	[0.0, 0.0, 0.0]
Data File	average size of data files (csv)^b^	309,999.4 (±4,314,537)	337,453.3 (±2,901,912)	532,226.8 (±3,595,252)	248,120.4 (±2,268,705)	[1,732.0, 7,017.0, 33,942.0]	[1,419.0, 6,046.5, 53,402.0]	[1,692.0, 10,398.0, 79,279.0]	[4,763.8, 28,315.0, 73,671.0]
	average size of data files(xls(x))^b^	426,555.6 (±2,755,034.2)	528,439.2 (±2,953,938)	360,737.8 (±2,050,485.3)	330,846.9 (±1,518,167.8)	[20,430.2, 30,511.0, 83,968.0]	[20,287.0, 45,568.0, 147,138.5]	[16,856.8, 45,056.0, 203,837.5]	[16,896.0, 34,462.0, 95,356.0]
	no. of rows (csv)^a^	3,845.2 (±50,528)	4,324.6 (±52,089)	6,221.6 (±55,637)	3,087.6 (±35,192.0)	[41.0, 85.0, 569.0]	[33.0, 79.0, 719.0]	[42.0, 147.0, 930.0]	[41.0, 118.0, 293.0]
	no. of columns (csv)^b^	23.3 (±340.0)	16.3 (±376.5)	23.7 (±524.6)	14.7 (±363.2)	[3.0, 7.0, 18.0]	[2.0, 4.0, 7.0]	[3.0, 6.0, 13.0]	[4.0, 11.0, 11.0]
	no. of rows (xls(x))	1,337.2 (±22,013.9)	409.4 (±10,184.4)	324.2 (±8,992.9)	1,105.0 (±16,615.8)	[26.0, 64.0, 141.0]	[64.0, 86.0, 122.0]	[19.0, 31.0, 52.0]	[20.0, 46.0, 176.0]
	no. of columns (xls(x))	29.8 (±397.2)	36.2 (±531.0)	23.8 (±155.0)	25.6 (±423.3)	[5.0, 9.0, 16.0]	[19.0, 19.0, 19.0]	[9.0, 12.0, 16.0]	[6.0, 10.0, 15.0]
	missing values ratio (csv)^a^	8.7 (±16.6)	7.2 (±19.1)	10.5 (±20.5)	13.0 (±13.6)	[0.0, 0.0, 11.3]	[0.0, 0.0, 0.0]	[0.0, 0.0, 11.7]	[0.0, 19.0, 19.8]

Quantiles values are reported in the [$x_{25}$, $x_{50}$, $x_{75}$] format, where $x_{25}$, $x_{50}$ and $x_{75}$ represent the 25th, 50th, and 75th quantile of a particular group's characteristic.

a Indicates statistically significant differences (p≤0.05) of pairwise comparisons across all four groups.

b Denotes cases for which statistical significant differences are observed between the values of groups 1 and 4 but not necessarily between the rest of pairwise comparisons.

### 6.2 Reuse Features

#### 6.2.1 Descriptive Statistics

Table 7 summarizes the values for all reuse features, grouped into the three types introduced earlier: repository, README file a.k.a. documentation, and data files. We annotate statistical significance determined by pairwise one-way ANOVA in the cases that the groups share a common standard deviation, otherwise we used Welch's t test.

**Table 7:** The Percentages with which the Most Frequent Programming Languages Are Met in Our Corpus

Language	%
Python	17.84
PHP	14.29
JavaScript	12.48
Java	9.40
HTML	6.63
C++	4.26
Jupyter Notebook	3.95
Ruby	3.63
R	3.50
None	3.572

The programming language of each data repository is determined based on the language that is used in the majority of its code files.

#### 6.2.2 Analysis of README Files

We selected the top ranked 20 repositories, according to our aggregated list of engagement metrics, for a manual analysis of their README files. These files provide a potential interesting source of information regarding the documentation indicators as discussed above.

We expand our analysis of reuse features manually to further include non-measurable elements identified in Table 1. Those that occur frequently could indicate useful areas of investment in regard to automation and tracking as they naturally come up in unstructured documentation of the most reused dataset repositories.

As shown in Table 8, 78.6% of the README files could be matched to an English language dictionary. Other languages were represented by below 2%. For 8% of the files we could not identify the language.

**Table 8:** Analysis Results of README Files

Characteristic	Mean (±SD)	Median
No. of headers	3.6 (±17)	1
No. of tables	0.08 (±0.97)	0
No. of images	0.684 (±3.9)	0
No. of text (words) (without code)	378 (±1,127%)	112
No. of code blocks	1.4 (±4.7)	0

We also analyzed the files manually to get a better understanding of those features that are not possible to assess automatically. We applied thematic analysis, taking the features from Table 1 as primary categories to code for in the sample repositories.

• links to basic concepts
• links to resources
• developer instructions/best practices
• installation and processing instructions
• mailing list/contact person/community
• description of purpose

As expected, none of these repositories seem to be personal but rather belong to large, often commercial, projects. For larger repositories representing projects the READMEs included links to external documentation, such as a project website. We included the content of these resources in our analysis of documentation practices if they were easily accessible and mentioned in the README.

## 7 Reuse Prediction

We created a model predicting a datasets likelihood to be reused based on these four groups of reuse. Our model uses features of repositories, README files, and data files to learn what makes a dataset reusable in this particular context. We propose an architecture based on the combination of feedforward architectures and bidirectional recurrent neural networks (RNNs) that seeks to predict the reuse group to which a data repository belongs. The reuse group is one of four, as explained in Section 6.

Let $d^{\left(r,f,t\right)}$ be a data repository, where *r*, *f*, and *t* are feature vectors that describe its general repository features (e.g., license and description), its enclosed data files, and the accompanying README file, respectively. We built a model that predicts the group y∈{1,2,3,4} to which $d^{\left(r,f,t\right)}$ belongs. Our end-to-end architecture consists of: (1) a feedforward architecture that processes the features associated with the README file, (2) a bidirectional RNN formed of gated recurrent units (GRUs) processing the enclosed data files, and (3) a similar bidirectional GRU coupled with a feedforward architecture that process the textual description $r^{x}\in r$ of $d^{\left(r,f,t\right)}$ and the rest of the general repository features *r*, respectively.

The more direct and accurate indication of actual dataset reuse that can be acquired, the more accuracy the prediction model can gain as it is limited to the engagement proxies from which we derive reuse probabilities.

### 7.1 Processing the Repository Features

We use a feedforward architecture to process the general features of each repository as these are presented in Section 5.2, except its textual description, $r^{\left(x\right)}$, for which we use a bidirectional GRU and its license, $r^{\left(l\right)}$, which is processed through a simple fully connected layer. The vector that is given as an input to the feedforward architecture is computed after we concatenate all the intermediate feature vectors that correspond to each separate general repository feature (except the textual description and the license). We use the real values for each of those constituent features, except the license for which we use one-hot encoded vectors.^28^ The length of the license vector equals the total number of different licenses in our dataset, including a None license entry for the data repositories without any license information. The size and age of each data repository are transformed to a logarithmic (To avoid zero values, we incremented each variable by one before computing its natural logarithm) scale before they are used in our model. The vector representation $\tilde{r}$ for the $r^{\left(a\right)}$, $r^{\left(s\right)}$, $r^{\left(i\right)}$, $r^{\left(f\right)}$, and $r^{n}$ features is computed by forward propagating as follows:

(Equation 1) $$\tilde{r}=\left[r^{\left(a\right)};r^{\left(s\right)};r^{\left(i\right)};r^{\left(f\right)};r^{\left(n\right)}\right],$$

where […;…] represents vector concatenation.

#### 7.1.1 Processing the Description

We used a bidirectional GRU to encode the information in the data repository's description $r^{\left(x\right)}$. Let ${\vec{h}}_{t_{x}}^{l},{\overset{\leftarrow }{h}}_{t_{x}}^{l}\in R^{m}$ be the aggregated output of a hidden unit of the forward and backward pass respectively at time step $t_{x}=1\ldots T$ and layer depth l=1…L. The vectors at zero layer depth, $h_{t_{x}}^{0}=W_{x\to h}x_{t_{x}}$, represent the tokens (i.e., words), $x_{1},\ldots ,x_{T}$, of x that are given to the network as input. The parameter matrix $W_{x\to h}$ has dimensions [|X|,m], where |X| is the size of the input dictionary (i.e., all the unique words that appear in the descriptions of the data repositories in our corpus). We initialized this matrix using GloVe embeddings^99^ and allowed the network to fine-tune it during training. At each time step *t*, ${\vec{h}}_{t_{x}}^{l}$ and ${\overset{\leftarrow }{h}}_{t_{x}}^{l}$ are computed as follows:

(Equation 2) $${\vec{h}}_{t_{x}}^{l}={\text{GRU}}_{x}\left({\vec{h}}_{t_{x}-1}^{l},h_{t_{x}}^{l-1}\right),$$

(Equation 3) $${\overset{\leftarrow }{h}}_{t_{x}}^{l}={\text{GRU}}_{x}\left({\overset{\leftarrow }{h}}_{t_{x}-1}^{l},h_{t_{x}}^{l-1}\right).$$

The context vector $h_{t_{x}}^{l}\in R^{2m}$ that encapsulates the information from both the forward and backward pass at each layer *l* and time step *t* is computed as $h_{t_{x}}^{l}=$ $\left[{\vec{h}}_{t_{x}}^{l};{\overset{\leftarrow }{h}}_{t_{x}}^{l}\right]$, where […;…] represents vector concatenation. Subsequently, the vector that encapsulates all the information from x, is computed by aggregating the hidden states of the two passes at their last processing time step (i.e., $t_{x}=T$ and $t_{x}=1$ for the forward and backward pass, respectively) of the topmost layer s.t. $\tilde{x}=\left[{\vec{h}}_{T}^{L};{\overset{\leftarrow }{h}}_{1}^{L}\right]$.

We compute the vector representation *r* of the general repository features of a data repository by incorporating the textual description and license to the rest of the general repository features as follows:

(Equation 4) $$r=\left(W_{l}r^{\left(l\right)}+W_{r}\tilde{r}+W_{x}\tilde{x}\right)\text{,}$$

where $W_{r}:R^{5}\to R^{m}$ and $W_{x}:R^{2m}\to R^{m}$ are biased linear mappings and $W_{r}:R^{E}\to R^{m}$ is an unbiased linear mapping.

### 7.2 Processing the Data File Features

Let $f=f_{1},f_{2},\ldots ,f_{F}:f_{j}\leftarrow \left[f_{j}^{\left(r\right)};f_{j}^{\left(c\right)};f_{j}^{\left(n\right)};f_{j}^{\left(s\right)}\right]\forall j\in \left[1,F\right]$ be the sequence of data files that exist in the data repository $d^{\left(r,f,t\right)}$ s.t. $f_{j}^{\left(r\right)}\geq f_{j+1}^{\left(r\right)}\forall j\in \left[1,F-1\right]$, where $f_{j}^{\left(r\right)}$, $f_{j}^{\left(c\right)}$, $f_{j}^{\left(n\right)}$, and $f_{i}^{\left(s\right)}$ are the respective number of rows, columns, missing values, and the size of each individual data file. Similarly to the case of the description, we use a bidirectional GRU at each time step, $t_{f}\in \left[1,F\right]$, of which we process a single data file. Consequently, the vector representation of the corresponding forward and backward pass, ${\vec{h}}_{t_{f}}^{l}$ and ${\overset{\leftarrow }{h}}_{t_{f}}^{l}$, respectively, are computed as follows:

(Equation 5) $${\vec{h}}_{t_{f}}^{l}={\text{GRU}}_{f}\left({\vec{h}}_{t_{f}-1}^{l},h_{t_{f}}^{l-1}\right),$$

(Equation 6) $${\overset{\leftarrow }{h}}_{t_{f}}^{l}={\text{GRU}}_{f}\left({\overset{\leftarrow }{h}}_{t_{f}-1}^{l},h_{t_{f}}^{l-1}\right).$$

Similarly to the case of the textual description, the vector that encapsulates all the information from the sequence of data files, f, is computed by aggregating the hidden states of the two passes at their last processing time step (i.e., $t_{f}=F$ and $t_{f}=1$ for the forward and backward pass, respectively) of the topmost layer s.t. $\tilde{f}=\left[{\vec{h}}_{F}^{L};{\overset{\leftarrow }{h}}_{1}^{L}\right]$.

### 7.3 Processing the README Features

Similarly to the case of the general repository features, we use a feedforward architecture to process the features associated with a README file of a data repository (cf. Table 3). Given $t^{\left(g\right)}$, $t^{\left(u\right)}$, $t^{\left(b\right)}$, $t^{\left(f\right)}$, $t^{\left(h\right)}$, $t^{\left(t\right)}$, $t^{\left(i\right)}$ ∈N, and $t^{\left(u\right)}\in 0,1$, represented as a two-dimensional one-hot vector, we compute a vector $\tilde{t}$ by concatenating the intermediate features as follows:

(Equation 7) $$\tilde{t}=\left[t^{\left(g\right)};t^{\left(u\right)};t^{\left(b\right)};t^{\left(f\right)};t^{\left(h\right)};t^{\left(t\right)};t^{\left(i\right)};t^{\left(u\right)}\right].$$

### 7.4 Predicting the Category of Reuse

After computing the *r*, $\tilde{t}$, and $\tilde{f}$ vector representations for the general repository, data files, and README features, respectively, the system projects the three modalities into a shared feature space. The resulting *context* vector $c_{d}$ for a data repository $d^{\left(r,f,t\right)}$ is computed as follows:

(Equation 8) $$c_{d}=\text{ReLU}\left(r+W_{t}\tilde{t}+W_{f}\tilde{f}\right),$$

where $W_{t}:R^{9}\to R^{m}$ and $W_{f}:R^{2m}\to R^{m}$ are biased linear mappings and $W_{r}:R^{E}\to R^{m}$ is an unbiased linear mapping. After computing $c_{d}$, our architecture predicts the category of reuse to which a data repository $d^{\left(r,f,t\right)}$ belongs by forward propagating a set of fully connected layers:

(Equation 9) $$\tilde{y}=\text{ReLU}\left(W_{d}^{\left(II\right)}\text{ReLU}\left(W_{d}^{\left(I\right)}c_{d}\right)\right),$$

where $W_{d}^{\left(I\right)}$ and $W_{d}^{\left(II\right)}:R^{m}\to R^{m}$ are biased linear mappings. The conditional probability distribution of the dataset reuse category to which $d^{\left(r,f,t\right)}$ belongs is represented with the softmax function over the total four categories of reuse:

(10) $$p\left(y|d^{\left(r,f,t\right)}\right)=\text{softmax}\left(W_{y}\tilde{y}\right),$$

where $W_{y}:R^{m}\to R^{4}$ is a biased linear mapping. Our model learns to make a prediction about the reuse category of a data repository by using the negative cross-entropy. During training and given a particular data repository $d^{\left(r,f,t\right)}$, the model predicts its category of reuse and it fine-tunes its parameters by seeking to minimize the negative log likelihood cost of the predicted probability distribution with respect to the actual reuse category of $d^{\left(r,f,t\right)}$.

### 7.5 Training Details

Both bidirectional RNNs used in our architecture (i.e., for processing the textual description and the data files of a given data repository) are implemented with 2 layers of 512 bidirectional GRUs. We included the |X|=5k more frequent tokens from the textual description. Occurrences of rare words in the text of a description are replaced by the special <rare> token.^100^^,^^101^ We augment each textual description with start-of-sequence and end-of-sequence tokens. For the purposes of the training and subsequent evaluation of our approach, we randomly split our dataset into training, validation and test, with respective portions of 70, 15, and 15.

We initialize all parameters with random uniform distribution between −0.001 and 0.001, and we use batch normalization before each non-linear activation function (i.e., ReLU) and after each fully connected layer.^102^ The training objective of our system is to minimize the mean of the negative log-likelihoods of the predictions for a mini-batch of 128 data repositories. The weights are updated using Adam^103^ with a learning rate of $10^{-3}$. An $l_{2}$ regularization term of 0.01 over each network's parameters is also included in the cost function.

To sidestep the uneven distribution of data repositories across the four categories of reuse (cf. Section 6.1.2), we opted to over-sample the minority classes during training. We found that this worked slightly better than weighting examples from the under-represented classes higher in the computation of the negative log-likelihoods.

### 7.6 Prediction of Data Repository Reusability

We describe the performance of our model and provide context to the limitations of the features we were able to use in this approach.

Combining all the available information (i.e., repository, data file, and README features) enables our predictive model to achieve its highest accuracy score of just under 60%. This means given the characteristics of a dataset, its repository, and its README file, we can predict with this accuracy whether it is going to be in the most reused group. Table 9 shows scores and gives an indication of the importance of the different feature types.

**Table 9:** Accuracy and ${\text{F}}_{1}$ Scores for Predicting the Reuse Category of the Data Repositories in the Validation and Test Set

System	Accuracy		${\text{F}}_{1}$	
	Validation	Test	Valididation	Test
Data files	49.10	49.11	38.92	38.46
Description	42.69	42.33	44.32	43.79
README	46.29	46.75	47.17	47.32
Repo	53.71	53.29	54.14	53.73
Repo + Description	53.72	53.58	54.31	54.31
Repo + README + Description	56.13	55.93	57.05	56.71
Repo + README + Description + Data Files	59.41	59.23	59.15	58.58

Table 10 shows the performance of our best performing system (i.e., the one capable of processing all the available features) across the four different classes of our classification task. We see that ${\text{F}}_{1}$ scores improve substantially for the groups that more distinctively represent reuse category (i.e., groups 1 and 4). Inspired by this result, we opt to group repositories that belong to groups 3 and 4 and repositories that are part of groups 1 and 2 into two different categories, a reused and a not-reused one, respectively. We measure the performance of our best performing system on this binary classification task.

**Table 10:** ${\text{F}}_{1}$ Scores of the Predictions of Our Best Performing System on the Test Set across the Four Different Reuse Categories of Classification Task

Class	Precision	Recall	${\text{F}}_{1}$	No. of Samples
Group 1	0.76	0.78	0.77	3,493
Group 2	0.40	0.27	0.32	1,646
Group 3	0.31	0.41	0.35	821
Group 4	0.48	0.63	0.54	568

The results are reported in Table 11, indicating that our model predicts the likelihood of a data repository not being reused with high confidence (in more than four out of five cases). Due a variety of external reasons that go beyond features that can be implicitly obtained via GitHub, accurately predicting that a data repository will be reused is a more challenging task. However, our model achieves a promising performance providing groundwork for further research in this area.

**Table 11:** ${\text{F}}_{1}$ Scores of the Predictions of Our Best Performing System on the Test Set in a Binary Classification Task for Reuse Predictions

Class	Precision	Recall	${\text{F}}_{1}$	No. of Samples
Not-reused (groups 1 and 2)	0.92	0.84	0.88	5,139
Reused (group 3 and 4)	0.56	0.74	0.63	1,389

Instances of data repositories that belong to groups 1 and 2 are considered as not-reused, whereas repositories that belong to groups 3 and 4 are considered as part of the reused class.

We use the features in Table 3 in the model, as these are provided by the GitHub API and tracked across the large number of dataset repositories we investigated. However, hypothetically many other indicators listed in Table 1 could be represented as part of this architecture if tracked across a large number of dataset repositories. This opens up a large space for both research in this area to develop the model further, but also shows how publishers and other data stakeholders could track reuse and impact.

## 8 Findings

The final step reflects upon the results of the analysis, including the prediction model to identify recommendations and areas of improvement. Here, we discuss these in the context of the GitHub Case Study.

### 8.1 Features of Popular Data Repositories

In our study, we looked at a large corpus of 1.4 million datasets using common structured formats, such as CSV and Excel. We structured our analysis in three parts: repositories (which are essentially folders of code, data, and other resources), documentation of repositories (README files), and the data files themselves. We clustered the repositories into four groups, with group 4 achieving highest reusability according to the indicators. We manually inspected the README files, which are written in free text to detect themes.

Most features show significant differences between all four groups. The features with no significance include the number of columns and rows of the xls(x) files as well as the number of columns in the CSV files.

As shown in Table 6, the size of the repository increases with higher reuse probability. The most reused datasets also seem to have more detailed README files. All README-related features show significant differences between the reuse groups, indicating higher complexity for the more reuse repositories in terms of their building blocks (more tables, images, links to other sources and code). The README files of the repositories from group 4 were also found to have more words in the files and to be significantly larger in size than in the other groups. They further contain more headers, which points to a higher degree of structure in the documentation. Repositories from the most reused group also tend to have more detailed and longer descriptions.

Furthermore, popular repositories show a higher number of closed issues and slightly more open issues, which confirms higher engagement.

We also tried to open the data files (using a standard library, in our case Pandas (https://pandas.pydata.org/)). The most reused group showed the lowest ratio of problematic files and can in that respect be considered to be more accessible and potentially of better quality. In our analysis, we also consider such aspects, for instance, missing values (see Table 6).

The age of a repository does not seem to be a strong indicator for reuse; the difference is limited to a standard deviation of 76 days (median). This means that older repositories, which could potentially have larger engagement metrics due only to their age, did not influence overall reuse ranking considerably. This gives us more confidence that our ranking is indeed a proxy of reuse and is not is just an artifact of age.

Looking at the number of rows in CSV files, the group with the highest reuse probability is more homogeneous, with a low standard deviation and the least number of rows in the data files, but with a high median in terms of file size. Comparing the number of rows and columns we assume that these indicators are not likely to be deterministic for reuse. The file size for the most reused group is smaller in contrast to group 3, which we hypothesize could be due to the added technical barrier of reusing large data files.

The ratio of data files in the repository decreases in the more reused groups. This might be due to larger repositories, containing more and different file types, including supporting material that facilitates the use of the repositories code and data. This could also partially explain the high ratio of missing values that can be observed in the data files of the more reused groups, since they might require tailored configuration setting for opening them (which in many cases are described in their supporting material). In that case our approach of opening them with the standard configuration of the Pandas library might not read the file structure correctly.

### 8.2 Predicting Reuse

We combined the characteristics just discussed in a proof-of-concept machine learning model that estimates dataset reusability. The results are useful in multiple ways and demonstrate the feasibility of the approach.

Repository features account for over 50% of accuracy in the prediction. To some degree this is due to the nature of the GitHub environment, which is used to publish and share, among other things, software and data. GitHub is not designed as a data portal, does not offer capabilities to search for datasets or engage with them outside of a repository. In the same time, the link between datasets and context for reuse could be observed in native data environments as well.^42^

The main value of our prediction work is in showcasing how machine learning could be used in practice, using GitHub as a case study. As such, it is meant as a prototype that is useful for other contexts from a modeling perspective. We believe the accuracy could substantially improve with a larger corpus of datasets and by further tailoring the architecture to the task at hand but this would need to be validated in future work.

We believe this case study provides initial evidence for how data publishers, portal owners, and other stakeholders could close the gap between principles and practice of data reuse, through the use of automatic tools to monitor reuse and explore which design decisions and capabilities make a difference. In our case study, we combined a several feature types—counts, ratios, binary categories, as well as short text snippets—and tied them together to represent a dataset and the environment in which it is published. Another added variable is the variation of tabular data files per repository, which reflect real-world datasets that likely have a range of characteristics, such as number of columns, rows, or missing values.

#### 8.2.1 Exemplary Recommendations for the GitHub Usecase

To summarize, our results suggest the following recommendations for dataset publishers on GitHub:

1. provide an informative short textual summary of the dataset
2. provide a comprehensive README file in a structured form and links to further information
3. datasets should not exceed standard processable file sizes
4. datasets should be possible to open with a standard configuration of a common library (such as Pandas)

Our machine learning model indicates that these three aspects directly impact on how reused the data are going to be.

In the following section, we discuss more general implications and lessons learned from the this work.

#### 8.2.2 Mapping Reuse Features to GitHub

Many of the reusability features compiled in Table 1 were not measurable in the GitHub Case Study. GitHub suggests some of them explicitly, such as license info, the owner or author of a dataset, the availability of code, a README and a repository description or the file format. Our results showed the importance of not just a minimum length README file, but of a structured description of the dataset and its repository, pointing to various aspects of summary representations and understandability. This also suggested the importance of connections, namely, being a contact point as well as links to the dataset being used elsewhere. Beyond that, connections could also be seen as access to richer types of context, external concepts, and the ability to ask questions through community engagement (discussion forums, FAQs with advice on data reuse and caveats, more extensive project documentation or methodology). Our results further suggest the importance of a processable size of a dataset, compatible with common libraries, which points to the importance of the feature prior reuse/advice on data reuse. In summary, if wide uptake and reuse is the goal of a data publisher on GitHub, datasets need to be accessible in terms of machine readability and format, and come with code as well as documentation to help users understand context, including advice on reuse.

Being able to measure the various reusability features enables us to study which ones are the most useful in a particular repository or portal context and consequentially to prioritize efforts toward those features that help people reuse datasets. On the one hand this exemplifies the limitations of our work (see Section 10), but on the other hand it facilitates an informed discussion about the features worth measuring, which we discuss in the next section.

## 9 Discussion

The lack of tools that support tracking and analyzing the usage of data throughout their publication cycle has been pointed out in the literature (e.g., in Allen and Hartland^70^). Our literature review depicts a huge space, within and across disciplines and domains, of guidelines, frameworks, and technologies that are expected to be instrumental to enable data reuse. We have shown that some of them can be mapped to an existing data publishing and sharing infrastructure, namely, GitHub. We believe there is a large design space to create tools that capture and use reuse indicators for increased transparency and accountability around datasets.

### 9.1 Revisiting Reuse Principles

The GitHub use case exemplifies the importance of measurable reusability features. While not all features mentioned in Table 1 are easily measured, an even smaller number are currently being logged by common data repositories. Given that the ability to track and measure is a prerequisite to determining which features are useful indicators for reuse, this should be considered early in repository development and setup.

Our findings suggest that features related to understandability should be prioritized. Actively supporting people in understanding and working with the data are critical for facilitating reuse. This includes providing data in a way consumers can easily try and work with: small datasets that are easy to process, example code, accessible and structured context and explanations, links to external sources, plus the support of engagement around the dataset. The literature has shown that social interaction, including the ability to ask questions about the data, supports reuse.^95^ Our findings further suggest that the level of detail and structure of the README, as well as to some extent of the dataset repository, influences the likelihood of reuse.

Given current limitations to quantify reuse features we believe the next step is to implement functionalities to track a larger number of features within a repositories context. For instance, this includes the impact of using common vocabularies, as recommended as part of the FAIR principles, on dataset reuse. We hypothesize that if a vocabulary is used by software the impact on reusability will likely be more significant. The impact of visual summary representations of the dataset or of individual columns on reuse likelihood would be an interesting area to explore, given the importance of structured documentation our results suggest. For other features, such as reporting methodology, representativeness or relationships between variables one might need to think about standardized ways of capturing such information, either from the dataset or from the creator, before being able to measure impact on reusability. Such efforts are realized in some domains (i.e., Gamble et al.^44^), but often result in extensive manual documentation.

### 9.2 Designing Documentation for Reuse

README files on GitHub are completely flexible, they have no required structure or content. Our findings showed most READMEs of the top ranked data repositories are structured via headers and categories and contain different content types. This points to the opportunity to create checklists or templates for data descriptions, which was contemplated previously in the literature,^21^^,^^22^ and encourage structure and a variety of building blocks, through an authoring tool (similarly as for data visualization or data-driven storytelling^104^). This could also be achieved by extending existing interfaces for metadata provisioning (for instance, in CKAN or any other repository software), but including a range of aspects that are not captured by existing metadata vocabularies, which are meant for machine consumption to enable search.^62^ Similar concerns around creating documentation for the purpose of reuse, without large overhead, apply for reuse of other digital artifacts (e.g., code components, design systems). Facilitating documentation to navigate uncertainty around datasets could help align the expectations of reusers with the actual utility of a dataset for their task.^3^^,^^57^

Extensive documentation has been proposed in various forms; for instance, in the form of checklists that are standard in safety critical domains.^22^ Recent calls for more comprehensive documentation practices in the machine learning community have resulted in an increasing interest in “datasheets” or similar concepts.^21^^,^^22^ This work aims to contribute toward consolidating these efforts by focusing on observable features and metrics that are likely to contribute to eventual reuse.

Narrowing the breadth of reusability features can support the creation of intelligent user interfaces that facilitate impactful documentation. This can be anywhere on the spectrum of simple checklists, to capturing the complexities of communicating study designs and methodological details using different media types, such as tutorial style videos or interactive environments exploring a datasets creation timeline in a virtual space.

### 9.3 Managing Reuse

Our prediction model offers a prototype tool that would enable dataset creators to get an idea of the expected reuse of a dataset before publishing. Ultimately this would allow a system to create tailored recommendations for reuse indicators to increase a dataset’s reusability by analyzing the files and documentation characteristics.

Our proposed tool, or a more sophisticated version, could be coupled with other approaches to help identify missing features and suggest improvements to both the dataset and the documentation. This could include measuring the completeness of the data, testing data validity,^105^ or semi-automatic support for dataset summary creation.^62^

We are aware that asking people to create more documentation comes at a cost. Parts of it could be generated automatically, but there are limitations.^106^ Our approach is different as it does not require data producers to change how they release datasets dramatically. We suggest mapping recommendations from the literature to actual capabilities of the system. We use existing engagement metrics to train a model that can identify the expected reusability of a dataset based on observable reuse features of a data repository and its environment. To improve the performance of the model, one could include a whole range of additional features, including information about the producers or the domain of the dataset as well as user activity logs.

### 9.4 Applying the Model in Different Contexts

While our prediction model is not out-of-the-box applicable to every dataset repository, its modular design provides its users with the necessary capacity to model alternative use cases. We present a set of modules for processing features of different nature ranging from tabular data (i.e., in Section 7.2) and markdown files, consisting of different structural sections, such as tables, URLs, and codeblocks (i.e., in Section 7.3) to combinations of textual data with other continuous and discrete variables (i.e., in Section 7.1.1). Furthermore, any feature in the proposed modules can be easily amended without any subsequent change in the formulation of the rest of the model's architecture. While each data source provides different features and reuse proxies, we believe that the plug-and-play design of our prediction system can simplify its adaption in other dataset-reuse-prediction tasks, once the input and output are properly determined.

## 10 Limitations

In this section, we discuss the limitations of this work regarding the dataset corpus and the GitHub use case, as well as the limited availability of measurable reuse metrics and reuse features.

### 10.1 Corpus

GitHub has primarily been developed as a platform to share and engage with code, not structured datasets. However, it is one of most widely used collaboration platforms in this space and our analysis showed the large amount of structured data published through GitHub. Kalliamvakou et al.^107^ found that GitHub is also used to archive data. Due to the nature of the platform it can be assumed that many datasets are connected to code that represents, while limited, a use case of data sharing and reuse on the web.

The dataset corpus reflects that GitHub repositories (as well as individual files) are restricted in size^31^; which means very large data files cannot be archived on the platform. This is also relevant for image- or audio-oriented datasets which tend to be large in size and are therefore unlikely to be stored on GitHub.

To obtain more evidence of the potential generalizability of the sketched approach, similar studies with other data portals or corpora would need to be conducted. While other platforms (e.g., Kaggle^32^ and Zenodo^33^) have different engagement metrics we believe that, dependent on data access, the approach can be applied to different contexts. This is an important direction for further research.

### 10.2 Reuse Metrics

We used engagement metrics and features characterizing the repository as proxies for the reuse indicators. We did not have access to the number of downloads per dataset via the GitHub API. Most data portals have this information readily available and adding to the model is straightforward. One can imagine other potential metrics as proxies for reuse indicators as well as different metrics for different types of reuse. This work provides a starting point to think about what we could predict and recommend if we start measuring and capturing reuse indicators more directly and for different contexts.

### 10.3 Features

Many of the reuse indicators from Table 1 could not be automatically measured in our use case analysis and not all might be measurable. There is a limited availability of automatically extractable features on GitHub and we hypothesize this to be the same for other online platforms used for data sharing and reuse. These reuse indicators might further look different or be more specific for certain domains and data types. For instance, the indicators for streaming data would likely include different elements.

We accessed the data files to understand the share of missing values, whether they can be opened with a standard configuration, and the number of rows and columns to get an understanding of the shape of the dataset corpus. We did not perform further analysis of the files themselves as this was outside the scope of this work. It would be interesting to run a similar style of study that links quality scores to reuse; a related work for Wikidata has shown how such scores for individual data records are impacted by who creates the data and how.^108^

When language was used as a feature, we restricted this to checking the language of the README file. While this is restrictive, we chose the English language as a feature because it was represented in over 70% of all README files. All other languages were represented with a significantly lower ratio, hence we excluded them from our feature list.

In terms of missing values, we focused on CSV files only. Table 6 does not show the ratio of missing values for XLS(X) files. This omission is due to the XLS(X) structure of separate sheets and different formatting options. As they do not conform to a regular structure, it is difficult to be properly understood by the default parameters of a widely used library, such as Pandas^34^ (which we used to determine the ratio).

The manual analysis of README files might be influenced by the fact that GitHub likely has a fairly technical target audience and the presentation of documentation could be tailored to their perceived needs.^107^^,^^109^ The original purpose of the dataset or repository might not be stated explicitly, even for well reused ones, as the “purpose” is implicit in the project that they are attached to.

Finally, not everything about a dataset can be captured, taking into account the complex and situational processes of its creation. However, we believe our work contributes to help focus automation efforts for the purpose of dataset reuse.

## 11 Conclusions

We presented a detailed compilation of reuse features from literature. To understand how they look like in data projects, we carried out a case study of structured datasets published and shared on GitHub. We analyzed the structure of the repository they sit in, their documentation and, to some degree, the data files themselves. We established a gap between the features associated to reusability in the literature and those that could be observed or collected on GitHub.

Some aspects of data work cannot be turned into indicators easily, for instance, methodology, ethical details, and even more the social processes and negotiations happening during data creation. Our recommendation for data publishers is to invest into meaningful indicators for those features that can be demonstrably linked to increased uptake. This could be built on by integrating functionalities that measure engagement with datasets in an automated way and recommend indicators that would increase reuse probability. This would allow authors to increase a dataset's potential for reuse before publication, focusing on not just the data but also on documentation and other potentially relevant features of a project. Our recommendation for data science experts, including community initiatives and standardization bodies, is to ground recommendations and guidance into capabilities and activities commonly occurring in data projects, similar to the mapping we have carried out in Section 5.

We would like to extend this work by looking at other online platforms for data sharing, such as from the data science community or a collection of widely reused research datasets.

In summary, we hope that this paper illustrates the challenges of preparing datasets for reuse and moves the discussion forward on helping give data providers concrete, measurable, and operational advice on how to make their datasets more reusable.

## 12 Experimental Procedures

### 12.1 Resource Availability

#### 12.1.1 Lead Contact

Paul Groth is the lead contact of this study and can be reached at: p.groth@uva.nl.

#### 12.1.2 Material Availability

The source code for the machine learning model used in this study can be obtained via this GitHub repository: https://github.com/laurakoesten/Dataset-Reuse-Indicators.

#### 12.1.3 Data and Code Availability

The data and code have been made public at: https://github.com/laurakoesten/Dataset-Reuse-Indicators, including data for all dataset repositories used in this work, and data used for training the model. In addition, the data can be found at: Koesten, Laura, Vougiouklis, Pavolos, Groth, Paul, & Simperl, Elena (2020). Dataset Reuse Indicators Datasets (Version 1.0) [Dataset]. Zenodo. http://doi.org/10.5281/zenodo.4015955.
